# The proteome of frozen-thawed pig spermatozoa is dependent on the ejaculate fraction source

**DOI:** 10.1038/s41598-018-36624-5

**Published:** 2019-01-24

**Authors:** Cristina Pérez-Patiño, Junwei Li, Isabel Barranco, Emilio A. Martínez, Heriberto Rodriguez-Martínez, Jordi Roca, Inmaculada Parrilla

**Affiliations:** 1 Department of Medicine and Animal Surgery, Faculty of Veterinary Science, University of Murcia, Murcia, Spain; 2 Department of Clinical & Experimental Medicine (IKE), Linköping University, Linköping, Sweden

## Abstract

The preservation of sperm functional parameters and fertility post-cryopreservation largely varies in the porcine, a species with a fractionated ejaculate. Although intrinsic individual differences have primarily been linked to this variation, differences in protein abundance among frozen-thawed (FT)-spermatozoa are far more relevant. This study, performed in two experiments, looked for proteomic quantitative differences between FT-sperm samples differing in post-thaw viability, motility, apoptosis, membrane lipid peroxidation and nuclear DNA fragmentation. The spermatozoa were either derived from the sperm-rich ejaculate fraction (SRF) or the entire ejaculate (Experiment 1) or from the first 10 mL of the SRF, the remaining SRF and the post-SRF (Experiment 2). Quantitative sperm proteomic differences were analysed using a LC-ESI-MS/MS-based SWATH approach. In Experiment 1, FT-spermatozoa from the SRF showed better preservation parameters than those from the entire ejaculate, with 26 *Sus scrofa* proteins with functional sperm relevance showing relative quantitative differences (FC ≥ 1.5) between sperm sources. In Experiment 2, FT-spermatozoa from the first 10 mL of the SRF and the remaining SRF were qualitatively better than those from the post-SRF, and 187 proteins showed relative quantitative differences among the three ejaculate sources. The results indicate that quantitative proteome differences are linked to sperm cryosurvival.

## Introduction

Improving fertility outcomes of frozen-thawed (FT) spermatozoa remains a pending challenge for some livestock species, including the porcine^1^. In spite of the valuable progress in cryopreserving boar spermatozoa in recent years^2^, parameters defining relevant post-thaw sperm attributes are still variable and affect fertility, which remains considerably lower for FT-semen compared to liquid-stored semen^3,4^. This status of variable cryosurvival impairs the efficient inclusion of FT-spermatozoa in commercial artificial insemination (AI)-programs^5^ and is not exclusive to pigs since it also occurs in other species, such as the ovine^6^ or humans^7^. However, it is especially relevant for porcine commercial husbandry, where the magnitude of such variability classifies AI-boars as either good or bad sperm freezers^8^, sometimes impairing the efficient AI-use of genetically superior boars.

The usually consistent variability in sperm cryosurvival among boars and among ejaculates within boars is *in praxis* compensated for by matching the numbers of cryosurviving FT-spermatozoa to those of the viable spermatozoa used in AI-doses of liquid stored semen, e.g., increasing the number of FT-spermatozoa per dose. This process obviously implies up to a 4-fold increase in the total number of FT-sperm per AI-dose^5^, thus consuming more spermatozoa per boar. This accommodating and inefficient practice does, moreover, not match the fertility of FT-spermatozoa to that of the liquid stored semen, nor minimizes the variability between boars/ejaculates in AI-fertility^9^. Roca *et al*.^10^ noted that *in vitro* fertility outcomes of FT-boar spermatozoa from semen samples showing bad sperm freezability were lower than those showing good sperm freezability, even after inseminations with similar numbers of cryosurvived spermatozoa. This background suggests that there may be putative differences in molecular arrangement affecting fertilizing capacity between the FT-spermatozoa from semen samples differing in sperm freezability. Since proteins are involved in most critical sperm functions, including fertilizing ability^11^, the present study attempts to clarify this issue by analysing the proteome of FT-spermatozoa with documented freezability. It is currently known that cryopreservation remodels the proteome of boar spermatozoa^12^, but it is yet unknown if the proteome of FT-spermatozoa varies with the source of the spermatozoa, i.e., derived from semen sources with clearly different sperm freezability.

The boar ejaculate is emitted in fractions, and the so-called sperm-rich fraction (SRF) and the post-SRF are the two main fractions^13^. Currently, pig AI-centres are moving from selectively collecting the SRF, by using the gloved-hand method, towards collection of the entire ejaculate (EE), using semi-automatic methods^5^. This change in method for ejaculate collection is mainly motivated by sanitary and labour cost reasons. However, it does not advance animal welfare and jeopardizes sperm cryosurvival, since the post-thawing functional attributes of spermatozoa from the EE are worse than those from the SRF^14,15^. The current study, split into two experiments, aimed therefore to compare the proteome of FT-spermatozoa derived from the SRF and the EE (Experiment 1) and to compare that of the ejaculate fractions with clear differences in sperm freezability^15,16^, specifically, the first 10 mL of the SRF, the remaining SRF and the post-SRF (Experiment 2).

## Results

### Sperm proteome profile

A total of 93,457 spectra corresponding to 16,777 distinct peptides and 1,157 proteins were identified when assuming an FDR ≤ 1% at the protein level. Of the latter, 673 belonged to *Sus scrofa* taxonomy. The complete list of the 1,157 sperm-proteins identified, including their unused score, UniProt accession number, protein name, species, % of sequence coverage and matched peptides, is provided in Supplementary Table S1. The SWATH approach allowed the quantification of 1,094 sperm-proteins, of which 670 belong to *Sus scrofa* taxonomy (Supplementary Table S2). All the quantified proteins were present in FT-spermatozoa from the different sources evaluated, specifically the EE, the entire SRF, the first 10 mL of the SRF, the remaining SRF and the post-SRF.

### Proteins with different relative abundance in FT-spermatozoa among semen sources

The results comparing the entire SRF and the EE and those comparing the different portions of the ejaculate (first 10 mL of the SRF, the remaining SRF and the post-SRF) are separately shown. In addition, the results of the post-thaw assessment of sperm attributes are also included.

#### Differences between FT-spermatozoa retrieved from the SRF or the EE

**Figure 1:**
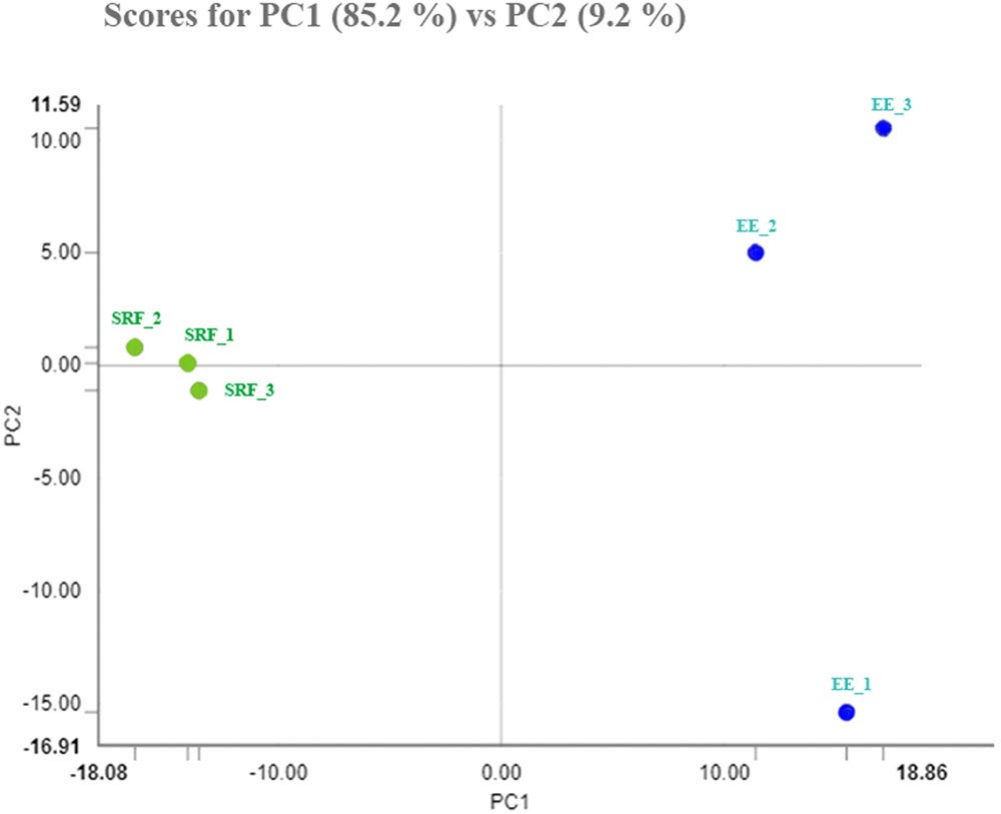
Principal Component Analysis (PCA) relative to the proteins quantified in frozen-thawed spermatozoa retrieved from the sperm rich fraction (SRF) of the ejaculate or from the entire ejaculate (EE). The points represent the three technical replicates for each sperm source and are based on the relative amounts quantified from each source.

**Figure 2:**
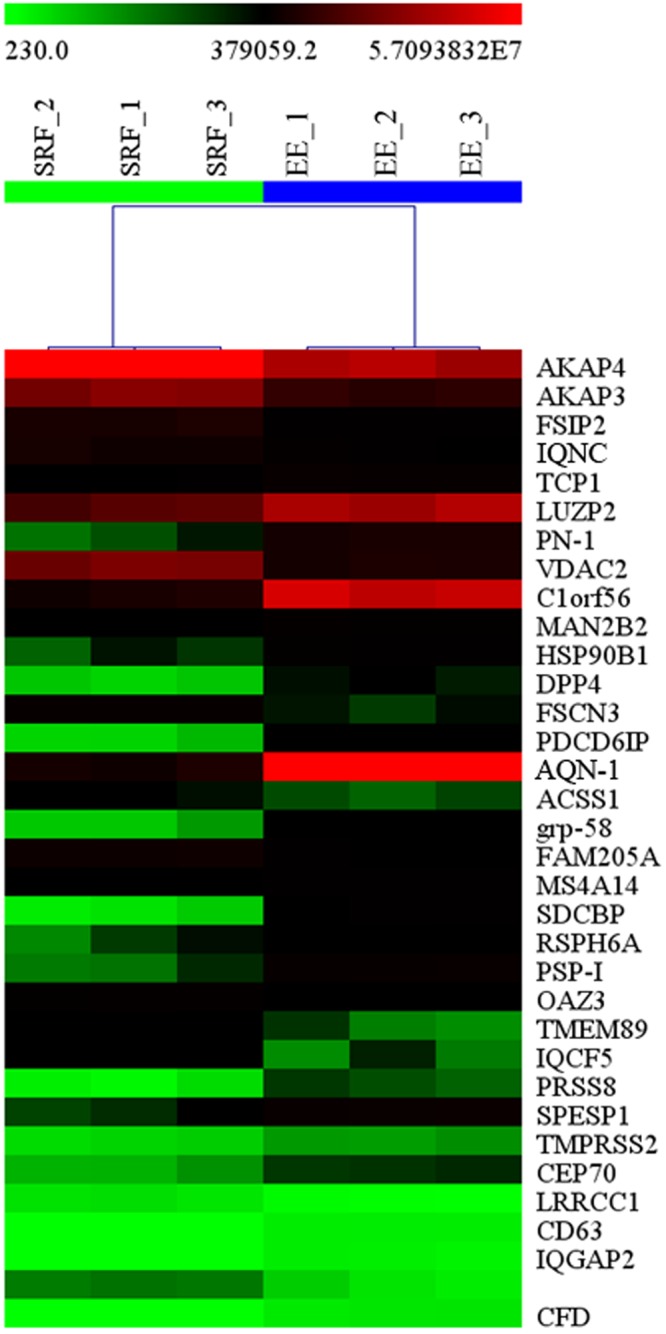
Heat-map with dendrograms representing the differentially abundant *Sus scrofa* proteins between frozen-thawed spermatozoa derived from either the sperm-rich ejaculate fraction (SRF) or the entire ejaculate (EE). The hierarchical clustering tree of sperm samples is shown at the top. The relative abundance level of each protein is shown on a colour scale from green (lowest level) to red (highest level).

**Figure 3:**
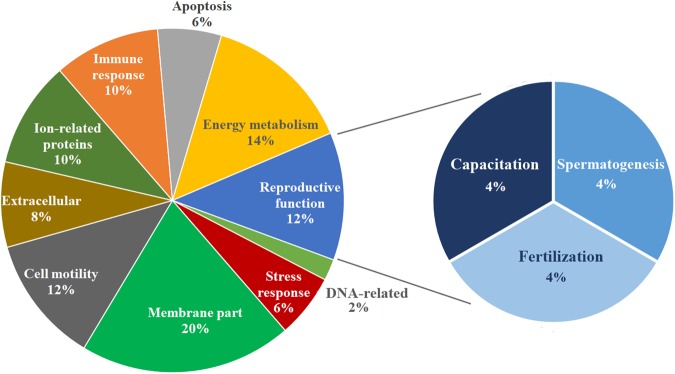
Functional distribution of *Sus scrofa* sperm proteins into the categories “sperm functions” and “reproductive functions” available on the UniProtKB/Swiss-Prot website (www.uniprot.org) and from DAVID Bioinformatics Resources 6.8 (https://david.ncifcrf.gov).

Concerning post-thaw sperm attributes, sperm source (P < 0.001) and boar (P < 0.01) influenced all sperm parameters evaluated 30 min after thawing, except for proportion of fragmented nuclear DNA. The interaction between the FT-sperm source and boar was not significant for any of the sperm attributes evaluated. Therefore, the data of the five boars were averaged for each sperm source. Post-thawing measured sperm attributes were better (P < 0.001) in spermatozoa from the SRF source than those from the EE (Table 1). Concerning the total FT-sperm proteins quantified, the two first components of the PCA explained 94.4% of total variance, and the PC1 (explaining 85.2% of total variance) discriminated between the three replicates of the SRF from those of the EE (Fig. 1). A total of 34 proteins belonging to *Sus scrofa* taxonomy showed quantitative differences (P < 0.01) between the FT-sperm from the SRF and those from the EE. The quantitative value of these proteins, after data normalization for each one of the two FT-sperm sources and the FC estimation of the groups after log2 transformation, is shown in Supplementary Table S3. Twenty-six of these 34 proteins showed an FC ≥ 1.50, and 11 of them were more abundant in the FT-spermatozoa retrieved from the SRF, whereas the other 15 were more abundant in those derived from the EE (Table 2). The heat-map of Fig. 2 showed that the three technical replicates of each FT-sperm source were grouped in a same cluster merged at a short distance; evidenced further by the dendrogram with a large distance between the cluster of SRF and that of EE. Regarding the allocation of the differentially quantified proteins into the functional categories contemplated in UniProt KB and the DAVID database and specifically focused on categories related to sperm and reproductive functions, most proteins showing quantitative differences were closely related to the cell membrane (20%, 10 proteins), energy metabolism (14%, 7), cell motility (12%, 6), and reproduction (12%, 6). The six differentially quantified proteins framed into reproductive function were equally distributed among spermatogenesis, sperm capacitation and fertilization (Fig. 3). Of note, some of the proteins showing quantitative differences were related to the immune response (10%, 5), apoptosis (6%, 3), the stress response (6%, 3) and DNA (2%, 1).

**Table 1:** Attributes (as percentage mean ± SEM) of frozen-thawed boar spermatozoa derived from the sperm rich ejaculate fraction or the entire ejaculate (15 ejaculates from 5 boars).

Sperm attributes (%)	Sperm source	
	Sperm rich ejaculate fraction	Entire ejaculate
Total motility	49.02 ± 1.79^a^	36.13 ± 1.16^b^
Progressive motility	40.93 ± 1.55^a^	31.87 ± 1.83^b^
Viability	54.05 ± 1.65^a^	41.53 ± 1.38^b^
Viable sperm with early apoptosis signs	4.17 ± 0.35^a^	10.74 ± 0.84^b^
Viable sperm with lipid peroxidation	4.76 ± 0.27^a^	12.00 ± 0.80^b^
Sperm with fragmented nuclear DNA	1.64 ± 0.24	2.07 ± 0.25

^a,b^P ≤ 0.001.

**Table 2:** Differentially abundant *Sus scrofa* proteins with a fold change (FC) ≥ 1.50 between frozen-thawed boar spermatozoa from the rich ejaculate fraction and the entire ejaculate.

Protein Name	Accession	Gene Name	FC
A-kinase anchoring protein 4	A0A286ZWH7	AKAP4	3.46
Fibrous sheath interacting protein 2	F1RYK8	FSIP2	2.43
Uncharacterized protein	A0A287AI93	IQCN	1.83
Nexin-1	Q8WNW8	PN-1	−4.49
Voltage-dependent anion-selective channel protein 2	F1S2F6	VDAC2	2.01
Chromosome 1 open reading frame 56	F1SSA1	C1orf56	−2.84
Endoplasmin	Q29092	HSP90B1 GRP94 TRA1	−2.38
Dipeptidyl peptidase 4	A0A2C9F3H7	DPP4	−2.07
Fascin	Q2I373	FSCN3	2.90
Programmed cell death 6 interacting protein	F1RRD6	PDCD6IP	−2.93
Carbohydrate-binding protein AQN-1	P26322	AQN-1	−3.80
Protein disulfide-isomerase	E1CAJ5	grp-58	−2.58
Family with sequence similarity 205 member A	I3L912	FAM205A	1.83
Uncharacterized protein	F1RT83	SDCBP	−4.20
Radial spoke head 6 homolog A	A0A287AGL6	RSPH6A	−1.54
Spermadhesin PSP-I	Q4R0H6	PSP-I	−3.34
Ornithine Decarboxylase Antizyme 3	I3LTK6	OAZ3	1.68
Transmembrane protein 89	F1SKK4	TMEM89	1.91
IQ motif containing F5	A0A287A4D1	IQCF5	1.73
Protease, serine 8	A0A286ZNI7	PRSS8	−2.69
Uncharacterized protein	A0A287B423	SPESP1	−2.99
Leucine rich repeat and coiled-coil centrosomal 1 protein 1	I3LBJ0	LRRCC1	1.83
Tetraspanin CD63	F1SPK8	CD63	−1.88
Ras GTPase-activating-like protein IQGAP2	K9J6M1	IQGAP2	−2.16
Uncharacterized protein	A0A286ZN09		1.82
Complement factor D preproprotein	B4F449	CFD	−3.18

#### Differences among FT-spermatozoa retrieved from three specific ejaculate portions

**Figure 4:**
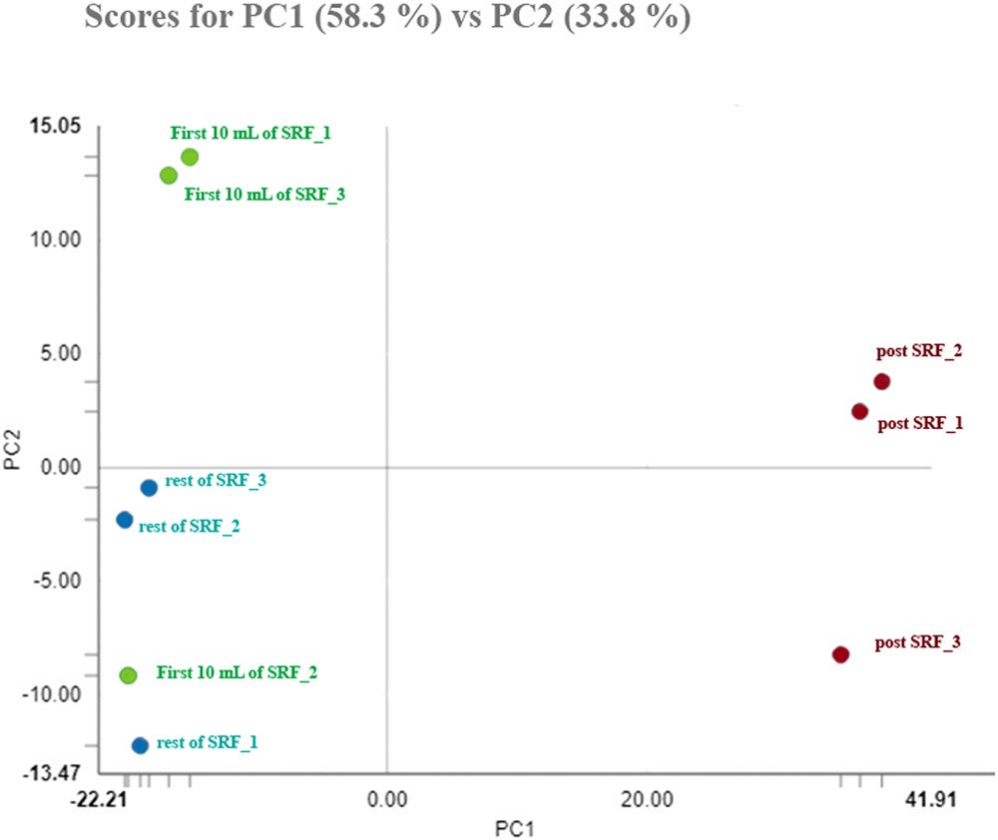
Principal Component Analysis (PCA) of proteins quantified in frozen-thawed spermatozoa retrieved from three identifiable portions of the pig ejaculate (the 10 first mL of the sperm-rich ejaculate fraction (SRF), the remaining SRF or the post-SRF). The points represent the three technical replicates for each sperm source and are based on the relative amounts quantified for each source.

**Figure 5:**
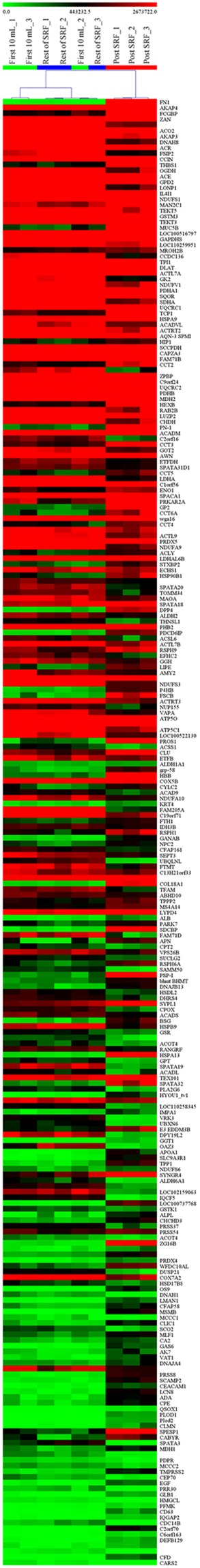
Heat-map with dendrograms representing the differentially abundant *Sus scrofa* proteins among frozen-thawed spermatozoa from three identifiable portions of the pig ejaculate (the 10 first mL of the sperm-rich ejaculate fraction (SRF), the remaining SRF or the post-SRF). The hierarchical clustering tree of sperm samples is shown at the top. The relative abundance level of each protein is shown on a colour scale from green (lowest level) to red (highest level).

**Figure 6:**
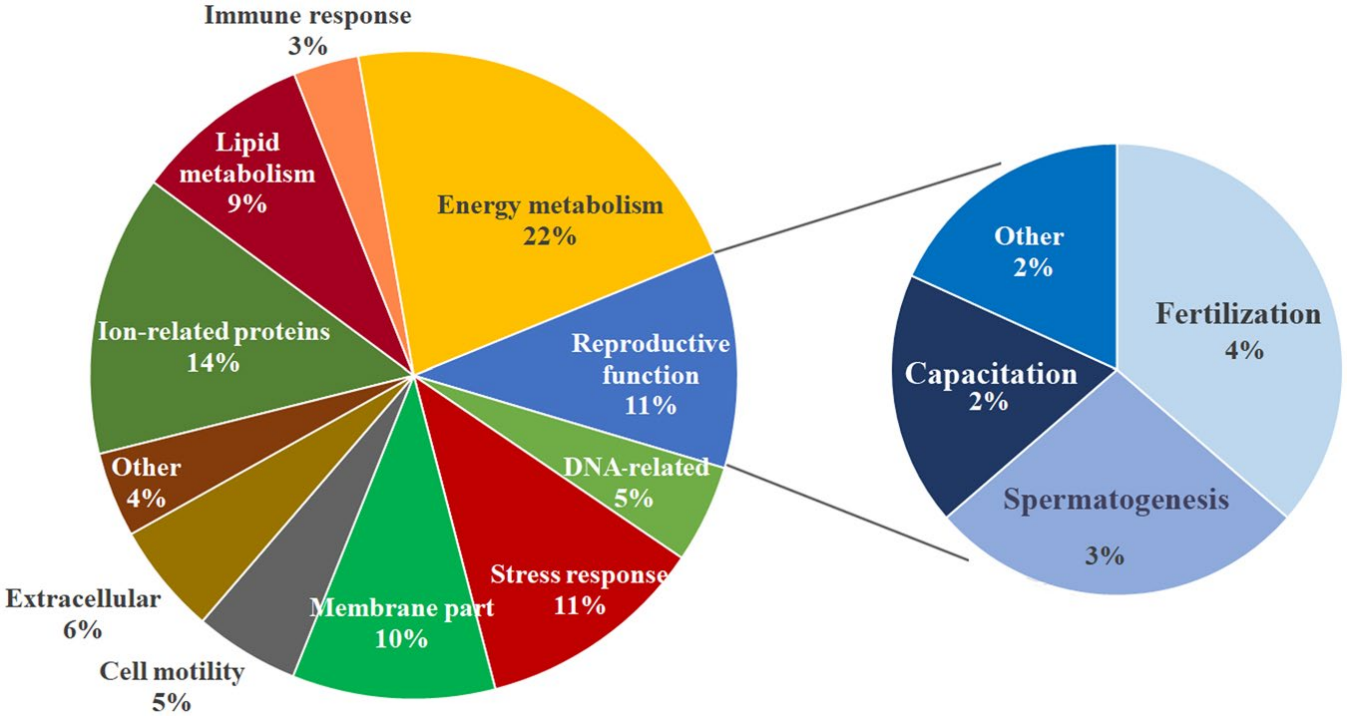
Functional distribution of *Sus scrofa* sperm proteins into the categories “sperm funcrions” and “reproductive functions” available on the UniProtKB/Swiss-Prot website (www.uniprot.org) and from DAVID Bioinformatics Resources 6.8 (https://david.ncifcrf.gov).

Concerning post-thaw sperm attributes, the sperm source and boar influenced (P < 0.01) all of the attributes, except for fragmented nuclear DNA. The interaction between the FT-sperm source and boar was not significant for any of the sperm attributes evaluated. Therefore, the data of the five boars were averaged for each sperm source. The post-thawing measured attributes were better (P < 0.01) in FT-spermatozoa from the first 10 mL of the SRF and the remaining SRF than those from the post-SRF (Table 3). Regarding the total FT-sperm proteins quantified, the two first components of the PCA explained 92.1% of the total variance. The PC1 (explaining 58.3% of total variance) best descriminate best among the three ejaculate portions/sources (Fig. 4). Accordingly, the FT-sperm samples were separated into two groups: the first one included samples from the first 10 mL of the SRF and the remaining SRF and the second group included samples from the post-SRF. A total of 257 proteins belonging to *Sus scrofa* showed quantitative differences (P < 0.01) among the FT-spermatozoa derived from the three ejaculate portions/sources. The relative amount of these proteins, following data normalization for each one of the three FT-sperm sources and the FC estimation of the groups after log2 transformation, is shown in Supplementary Table S4. A total of 187 of the differentially quantified proteins showed an FC ≥ 1.50 (Supplementary Table S5), most of them differing between FT-spermatozoa from the post-SRF and those from the two-other ejaculate portion/sources. Specifically, 173 FT-sperm proteins quantitatively differed between the post-SRF and the first 10 mL of SRF, while 165 proteins differed between the post-SRF and the remaining SRF. In addition, 90 FT-sperm proteins from the post-SRF were quantitatively higher than in the first 10 mL of the SRF, while 103 FT-sperm proteins from the post-SRF were quantitatively higher compared to the remaining SRF. The heat-map in Fig. 5 showed that the three technical replicates of FT-spermatozoa from the post-SRF were clustered together, clearly separated from those of the FT-spermatozoa derived from either the first 10 mL of SRF or the remaining SRF, grouped into a same cluster. Regarding the allocation of the differentially quantified proteins into the functional categories in UniProt KB and the DAVID database and specifically focused on those related to sperm and reproductive functions, most of the proteins were related to energy metabolism (22%, 66 proteins), ions (14%, 43), stress response (11%, 35), and reproduction (11%, 33). Most of the differentially quantified proteins framed into reproductive function were related to fertilization (4%, 12), spermatogenesis (3%, 11) and sperm capacitation (2%, 6) (Fig. 6). Some of these differentially quantified proteins were also related to the cell membrane (10%, 31), lipid metabolism (9%, 27), cell motility (5%, 16) and DNA (5%, 15).

**Table 3:** Attributes (percentage mean ± SEM) of frozen-thawed boar spermatozoa retrieved from three clearly identifiable ejaculate fractions (the first 10 mL of sperm-rich ejaculate fraction [SRF], the remaining SRF and the post-SRF; 15 ejaculates from 5 boars).

Sperm attributes (%)	Sperm source		
	First 10 ml of SRF	Rest of SRF	Post SRF
Total motility	53.67 ± 1.98^a^	47.40 ± 2.35^a^	20.87 ± 1.50^b^
Progressive motility	44.33 ± 1,74^a^	40.87 ± 2.27^a^	15.73 ± 0.84^b^
Viability	58.50 ± 1.99^a^	52.55 ± 2.54^a^	26.09 ± 1.88^b^
Viable sperm with early apoptosis signs	4.26 ± 0.47^a^	4.69 ± 0.44^a^	19.32 ± 1.36^b^
Viable sperm with lipid peroxidation	3.73 ± 0.36^a^	4.69 ± 0.70^a^	16.63 ± 1.27^b^
Sperm with fragmented nuclear DNA	1.62 ± 0.24	1.82 ± 0.25	1.78 ± 0.22

^a,b^P ≤ 0.01.

## Discussion

The results of the present study showed quantitative differences of proteins with relevance for sperm function among FT-spermatozoa. The results showed differences in freezability and sperm cryosurvival from different portions/fractions of the pig ejaculate. The results highlight that the spermatozoa derived from the EE would be less functional post-thaw than those retrieved from the SRF because of the differential relative abundance of some proteins, a matter which could seriously impair sperm fertilizing capacity.

The results from the post-thaw sperm confirm previous reports, demonstrating that boar spermatozoa from the SRF cryosurvive better than those retrieved from the EE^14,15^. Cryosurviving spermatozoa derived from the EE showed higher proportions of dysfunctions (lower sperm motility and higher rates of apoptosis and membrane lipid peroxidation) compared to FT-spermatozoa derived from the SRF. Molecular derangement, involving proteins, probably underlies these differences in sperm freezability. Subtle changes in relative protein abundance may compromise the reproductive performance of spermatozoa since proteins are involved in membrane remodelling, capacitation, oocyte zona binding, acrosome reaction and fusion to the oolemma^17^. Moreover, sperm proteins, together with those of the seminal plasma, play a critical role in the immunological reaction of the internal sow genital tract tissues after insemination^18^.

A total of 26 proteins encoded by *Sus scrofa* with a relationship to sperm function were proportionally different, with an FC ≥ 1.5, between FT-spermatozoa retrieved from the SRF and those from the EE. Eleven of these proteins, including two still uncharacterized by current databases, were most abundant in FT-spermatozoa from the SRF, which had the best sperm attributes post-thaw. Many of these proteins are structural components of the sperm tail, playing essential roles in the activation of the flagellum^19–21^. A-kinase anchoring protein (AKAP)-4, the most abundant cytoskeletal glycoprotein of the sperm fibrous sheath, Fibrous sheath interacting protein 2, Fascin, Ornithine decarboxylase antizyme 3 and Leucine rich repeat and coiled-coil centrosomal protein 1 were among the detected proteins. A lower abundance of these proteins could indicate damage in the tail and/or the mitochondrial sheath of spermatozoa and would explain why FT-spermatozoa from the EE have decreased motility parameters than spermatozoa derived from the SRF. Similarly, the higher abundance of the other four proteins could also clarify the improved functionality shown by FT-spermatozoa from the SRF. In this sense, the mitochondrial outer membrane porin protein voltage-dependent anion-selective channel protein 2 (VDAC2) is considered a positive biomarker of boar sperm freezability^22^, actively participating in the regulation of sperm mitochondrial function^23^. Family with sequence similarity 205-member A (FAM205A) is overexpressed in subpopulations of ejaculated human viable spermatozoa with low nuclear DNA fragmentation^24^. Finally, the other two more abundant proteins in FT-spermatozoa from the pig SRF, specifically the Transmembrane protein 89 and IQ motif containing F5, are proteins of the sperm plasma membrane involved in membrane stabilization and permeability regulation^25^.

Fifteen proteins were more abundant in FT-spermatozoa retrieved from the EE. Such relatively higher abundance could explain why FT-spermatozoa from the EE were less functional than those retrieved from the SRF. For instance, one of these proteins, the dipeptidyl peptidase IV (DPP-IV or CD26), a mitochondrial-associated protein^26^ related to sperm motility^27^, could be involved in inducing premature acrosome reaction when present in excess on the sperm surface. This protein is transferred to the sperm surface from sperm-binding seminal plasma vesicles^28^, and studies clearly demonstrated that human and bovine spermatozoa reach acrosome reaction if cultured with seminal plasma vesicles rich in DPP-IV^29,30^. Endoplasmin, better known as tumour rejection antigen gp96, a chaperone involved in the rebuilding of the sperm surface during capacitation^31^, was more abundant in sperm that underwent stress conditions such as cryopreservation^32^. In addition, endoplasmin was also comparatively more abundant in cryopreserved bull spermatozoa with low motility parameters^33^. Spermadhesin AQN1, a seminal plasma protein that binds to the sperm plasma membrane over the acrosomal domain during ejaculation, is involved in the formation of the oviductal sperm reservoir^34–36^. It is maintained in this location before capacitation, to disappear once spermatozoa are capacitated^37,38^. However, many spermatozoa surviving cryoinjury often exhibit the phenomenon named “cryocapacitation”, characterized by capacitation-like changes in sperm membrane phospholipids^39,40^. In these “cryocapacitated” spermatozoa, the AQN1 remains present in the sperm surface^35^. The sperm equatorial segment protein 1 (SPESP1), a protein involved in sperm-oocyte binding^41^, is particularly exposed in acrosome-reacted spermatozoa^42^, being found comparatively more abundant in cryopreserved than in fresh pig spermatozoa^12^. In view of this, a larger amount of SPESP1 would suggest the presence of a larger number of acrosome-reacted spermatozoa, cells that would hardly fertilize oocytes following insemination.

The higher abundance of some of these 15 proteins in FT-spermatozoa retrieved from the EE would entail that their fertilizing capacity could be compromised. For instance, Nexin 1, Spermadhesins PSPI, Tetraspanin CD63 (CD63), Complement Factor D (CFD) and Ras GTPase-activating-like protein IQGAP2 (IQGAP2) have been related to fertility losses when found comparatively more abundant in either spermatozoa or in seminal plasma. A higher abundance of PSPI and Nexin 1 was related to lower fertility outcomes in artificially inseminated sows^43,44^. Similarly, CD63, CFD and IQGAP2 were most abundant in fresh human spermatozoa with poor embryo developmental capacity after ICSI^45,46^. Other of the more abundant proteins found in FT-spermatozoa retrieved from the EE are related to the immune response either through complement and T-cell activation or cytokine modulation, specifically Syndecan binding protein (also named Syntenin, SDCBP), the DPP-IV and the programmed cell death 6 interacting protein^47–49^. Their higher abundance could make FT-spermatozoa from the EE particularly susceptible to phagocytosis once inseminated in the internal genital tract of the sow^50,51^. The consequences on FT-sperm performance by the four remaining more abundant proteins is less clear. The higher abundance of protein disulfide-isomerase could be related to cryocapacitation^52^. Radial spoke head 6 homolog A (RSPH6A), a structural protein of the sperm fibrous sheath that is involved in sperm motility^53^, was more abundant in FT- than fresh ovine spermatozoa^6^. Protease serine 8 is an enzyme involved in semen coagulation^54^, and chromosome 1 open reading frame 56 was recently identified in boar sperm chromatin^55^. Their relationship to FT-sperm fertilizing capacity requires further studies.

It was interesting to note the conspicuous relationship of many of the above-mentioned abundant proteins with the seminal plasma. However, whereas none of the more abundant proteins in the FT-spermatozoa from the SRF were identified in the seminal plasma of this fraction of the ejaculate, 11 of the 15 most abundant proteins in the EE-derived FT-spermatozoa were present in the seminal plasma^44^. These findings would indicate that most of the more abundant proteins in the FT-spermatozoa from the EE would come from the seminal plasma, binding to the sperm surface either during ejaculation or in overnight storage before freezing *ex situ*. Moreover, as these proteins were also more abundant in the seminal plasma from the post-SRF^56^, it is reasonable to consider they could primarily bind to the spermatozoa present in the post-SRF fraction. This assumption would also explain why there were not quantitative changes between the FT-spermatozoa retrieved from the SRF (the first 10 mL of the SRF vs the remaining SRF). The assumption would suggest that the binding of the proteins to the spermatozoa had already occurred during ejaculation when in contact with seminal plasma. Spermatozoa of the three main, identifiable, ejaculate fractions, specifically the first 10 mL of the SRF, the remaining SRF and the post-SRF, were separately cryopreserved and the proteome of FT-spermatozoa was evaluated to clarify the above assumptions.

The post-thaw sperm attributes agree with those previously reported by Alkmin *et al*.^14^ and Li *et al*.^15^, confirming that spermatozoa derived from the post-SRF were those with the worst freezability. The results prove that distinguishable sperm populations with clear differences in freezability are present in a single porcine ejaculate, suggesting biological reasons for the presence of a fractionated ejaculate in this species. Taken together, the results indicate that the worse freezability of spermatozoa from the EE compared to those from the SRF would be due to the negative concerted contribution of spermatozoa and seminal plasma from the post-SRF in building the EE. The clear quantitative differences in the proteome between the FT-spermatozoa from the post-SRF fraction and those from the other two ejaculate fractions could explain the poor functionality of FT-spermatozoa from the post-SRF source, as they showed a relative overabundance of many proteins directly or indirectly involved in sperm dysfunctionality. As expected, the 15 proteins that were more abundant in the FT-spermatozoa from the EE than in the SRF were among the most abundant proteins in the FT-spermatozoa from the post-SRF. Most of the more abundant proteins in the FT-spermatozoa from the post-SRF were previously identified as abundant in its seminal plasma^56^. This scenario would also support the notion that seminal plasma proteins bind most likely to the spermatozoa during ejaculation rather than during overnight *in vitro* sperm storage, a step in cryopreservation.

In conclusion, the present study showed that relative differences in abundance of specific proteins could explain why spermatozoa retrieved from the EE were less functional post-thaw than those derived from the SRF, a matter with clear biological and practical implications. The decreased functionality of the FT-spermatozoa derived from the EE could be caused by the negative contribution of the FT-spermatozoa from the post-SRF, coated with several seminal plasma proteins whose overabundance could negatively influence freezability and sperm cryosurvival. These results should be taken into careful consideration by technicians responsible for collecting and cryopreserving porcine ejaculates, to the extent that they showed that FT-spermatozoa from the EE were less functional than those from the SRF.

## Methods

### Boars, ejaculates and sperm sources

All procedures involving boars and semen samples were performed following international guidelines (Directive 2010/63/EU) and were approved by the Bioethics Committee of the University of Murcia (research code: 639/2012). The chemicals used for elaborate semen extenders and the fluorescent probes used for sperm evaluation were purchased from Sigma-Aldrich Co. (St. Louis, MO, USA).

Five healthy, young (12- to 18-months-old), sexually mature and fertility proven boars (two Large White, two of Duroc and one of Landrace breeds) were used as semen donors. The boars were housed in a commercial AI-centre (Topigs Norsvin España, Madrid, Spain) under controlled environmental conditions (16 h of light and 15–25 °C) and subjected to regular ejaculate collections (two ejaculates per week). A total of 15 ejaculates (three per boar) were manually collected using the gloved-hand method. All the ejaculates met the minimum requirements stablished by the AI-centre for the preparation of insemination doses, specifically more than 200 × 10^6^ spermatozoa/mL with 75% and 80% of spermatozoa displaying normal motility and morphology. The ejaculates were collected in three separate fractions, specifically the first 10 mL of the SRF, the remaining SRF and the post-SRF. Volumes of the first 10 mL of the SRF and the remaining SRF were proportionally mixed to generate semen samples simulating entire SRFs. Similarly, volumes of the three ejaculate fractions were mixed for generating semen samples simulating EEs. The five resulting semen samples of each ejaculate were extended (1:1 vol:vol) in Beltsville Thawing Solution (BTS), cooled at 17 °C, placed into a Styrofoam box and transported to the Andrology Laboratory of the Veterinary Teaching Hospital (VTH) of the University of Murcia (trip < 2 h). Once in the VHT, the BTS-extended semen samples were stored overnight at 17 °C to be frozen the next morning.

### Semen cryopreservation and post-thaw sperm evaluation

Semen samples were frozen using the straw freezing protocol described by Alkmin *et al*.^14^. Briefly, semen samples were centrifuged (2,400 *xg* for 3 min; Megafuge 1.0 R, Heraeus, Hanau, Germany), and the resulting sperm pellets were extended to 1.5 × 10^9^ spermatozoa/mL in a Tris-citric acid-glucose extender supplemented with egg yolk (80:20, vol:vol). Then, the extended spermatozoa were cooled to 5 °C and re-extended to 1.0 × 10^9^ spermatozoa/mL in the same extender supplemented with 9% glycerol and 1.5% Equex STM (v:v) (Nova Chemical Sales, Scituate, MA, USA). The spermatozoa were then packed into 0.5 mL polyvinyl chloride French straws (Minitüb, Tiefenbach, Germany) and frozen on a metal rack 3 cm above liquid nitrogen (LN_2_) for 20 min (Freezing unit, Minitüb). After being stored in an LN_2_-tank (GT40, Air Liquide, Paris, France) for at least a week, the straws (two per sperm sample/source) were thawed in a circulating water bath at 37 °C for 20 s, the contents were extended in BTS (1:1, vol:vol) and the mixture was incubated at 37 °C for 30 min before sperm analyses.

Post-thaw total and progressive sperm motility was evaluated using a computer-assisted sperm analyser (ISASV1^®^ CASA, Proiser R + D, Paterna, Spain) and sperm viability was evaluated using a BD FACS Canto II flow cytometer (Becton Dickinson & Company, Franklin Lakes, NJ, USA) after labelling the spermatozoa (100-µL with 3 × 10^6^ spermatozoa) with 3 µL Hoechst 33342 (H-42; 0.05 mg/mL in PBS), 2 µL propidium iodide (PI, 0.5 mg/mL in PBS), and 2 µL fluorescein-conjugated peanut agglutinin (PNA-FITC, 100 µg/mL in PBS). Viable sperm were categorized as those that were H-42 positive, PI negative and PNA-FITC negative. Protocols are described at length in Li *et al*.^15^.

Further functional attributes of cryosurvived spermatozoa were cytometrically evaluated. The early events of apoptosis and membrane lipid peroxidation (100-µL with 2 × 10^6^ spermatozoa labelled with 2 µL H-42 and 10 µL of PI) were evaluated using the fluorescent probes Annexin V-FITC (3 µL) and BODIPY 581/591 C11 (2.5 µL), respectively^57^. Viable spermatozoa (H-42 positive and PI negative) showing either early signs of apoptosis (Annexin V-FITC positive) or membrane lipid peroxidation (BODIPY positive) were recorded. Lastly, nuclear DNA fragmentation was assessed using the Sperm-Sus-Halomax^®^ kit (Halotech DNA SL, Madrid, Spain) following the method reported by Alkmin *et al*.^58^.

### Sperm proteomics

The proteomics analyses were carried out in the Proteomics Unit of the University of Valencia, Valencia, Spain (member of the PRB2-ISCIII ProteoRed Proteomics Platform).

#### Preparation of sperm samples

Three straws for each one of the 75 sperm sample/source (five semen samples of each one of the three ejaculates from each of the five boars) were thawed a 37 °C and their contents were centrifuged at 600 *xg* for 20 min at room temperature (rt) (Megafuge 1.0 R, Heraeus) on a Percoll monolayer gradient (45% in PBS [v:v], Percoll^®^ P4937; Sigma-Aldrich Co.) to separate the spermatozoa from other putative cells and debris, including egg-yolk remnants. The sperm pellets were extended in PBS (1:10, v:v) and centrifuged again (300 *xg*, 10 min at rt). Then, the resulting sperm pellets were extended in PBS to a concentration of 1,000 × 10^6^ sperm/mL, and stored in cryotubes (2 mL Cryogenic vial, Fisher Scientific, Madrid, Spain) that were preserved at −80 °C (Ultra Low Freezer; Haier, Schomberg, Ontario, Canada) until protein analysis.

During proteomics analysis, sperm samples were thawed at room temperature and the fifteen sperm samples of each one of the five semen sources (first 10 mL of the SRF, the remaining SRF, the entire SRF, the post-SRF and the EE) were pooled, generating five single sperm pools (one pool per source). Each sperm pool was in turn split into three aliquots generating three technical replicates per sperm sample. Thus, a total of 15 sperm samples were analysed.

#### Protein extraction

Sperm samples were centrifuged at 14,000 *xg* for 10 min (Eppendorf 5424 R, Eppendorf AG, Hamburg, Germany) and the supernatant was discarded. The total protein from the resulting sperm pellets was extracted using 200 µL of UTC buffer [7 M Urea, 2 M thiourea, 4% 3-[(3-cholamidopropyl) dimethylammonio]-1-propanesulfonate, (CHAPS)], supplemented with protease inhibitor cocktail (1%, v:v), followed by vigorous stirring (one hour at 5 °C). The concentration of extracted protein was quantified by RC_DC Lowry (Bio-Rad, Richmond, CA, USA) following the manufacturer’s instructions. Then, a total of 30 µg of final protein extract was taken for one dimensional sodium dodecyl sulfate-polyacrylamide gel electrophoresis (1D SDS-PAGE) loading to remove the remaining UTC and other interferences for tandem mass spectrometry (MS/MS) analysis.

#### SWATH analysis

In-gel digestion processing: The 1D SDS-PAGE portion containing proteins was digested at 37 °C using 500 ng of sequencing grade trypsin (V511, Promega Co., Madison, WI, USA) following the protocol used by Shevchenko *et al*.^59^. The trypsin digestion was stopped with 10% trifluoroacetic (TFA) and the supernatant, containing the non-extracted digests, was carefully removed, leaving behind the sliced gels in the Eppendorf tube. For peptide extraction, 200 µL of pure acetonitrile (ACN) was then added, followed by incubation at 37 °C in a shaker for 15 min. The new supernatant containing the peptide mixture was carefully withdrawn and dried in a speed vacuum (ISS 110 SpeedVac System, Thermo Savant, ThermoScientific, Langenselbold, Germany) for 20 min and then re-suspended in 25 µL of 2% ACN and 0.1% TFA.

Liquid chromatography and tandem mass spectrometry (LC-MS/MS) analyses: For the spectral library acquisition, a mixture of all digested samples (2 µL of each sample) was examined by liquid chromatography (LC) using a NanoLC Ultra 1D plus Eksigent (Eksigent Technologies, Dublin, CA, USA), which was directly connected to an AB SCIEX TripleTOF 5600 mass spectrometer (AB SCIEX, Framingham, MA, USA) in direct injection mode. Five µL of the digested sample was loaded on a trap NanoLC pre-column (3 μm particles size C18-CL, 350 µm diameter × 0.5 mm long; Eksigent Technologies) and desalted with 0.1% TFA at 3 µL/min for 5 min. Then, the peptides were separated using an analytical LC column (3 μm particles size C18-CL, 75 µm diameter × 12 cm long, Nikkyo Technos Co®, Tokyo, Japan) equilibrated in 5% ACN and 0.1% formic acid (FA) (Fisher Scientific). Peptide elution was performed by applying a linear gradient from 5% to 35% of ACN containing 0.1% FA at a constant flow rate of 300 nL/min over 180 min.

The TripleTOF was operated in data-dependent mode, in which a time of flight (TOF) MS scan occurred from 350 to 1250 m/z, accumulated for 250 ms TOF followed by 150 ms TOF with the same scan range for MS. The 25 most abundant multiply charged (2+, 3+, 4+ or 5+) precursor peptide ions were automatically selected. Ions with 1+ and unassigned charge states were rejected from the MS/MS analysis. The rolling collision energy equations were automatically set by the instrument according to the equation |CE| = (slope) × (m/z) + (intercept) with Charge = 2; Slope = 0.0575 and Intercept = 9.

#### LC-SWATH-MS acquisition

To determine quantitative differences between the SRF and the EE and among the first 10 mL of the SRF, the remaining SRF and the post-SRF, the sequential window acquisition of all theoretical spectra (SWATH) analysis of individual sperm samples was performed following the procedure described by Perez-Patiño *et al*.^56^, tuning the TripleTOF 5600 (AB SCIEX) as described by Gillet *et al*.^60^ for SWATH-MS-based experiments. In this way, 5 µL of one of the three technical replicates from each sample was randomly loaded onto a trap column (NanoLC Column, 3 µm C18-CL, 75 μm × 15 cm; Eksigent Technologies) and flushed for 5 min with 0.1% TFA at 3 µL/min to remove salts. Peptide separation was achieved using an analytical column (LC Column, 3 µm C18-CL, 75 µm × 12 cm, Nikkyo Technos Co®) equilibrated in 5% ACN and 0.1% FA, eluted with a linear gradient from 5 to 40% ACN for 90 min at a flow rate of 300 nL/min. The analysis of eluted peptides was carried out in the spectrometer nanoESI-qQTOF (SCIEX TripleTOF 5600) and the TripleTOF operated in SWATH mode, in which a 0.050 s TOF MS scan from 350 to 1250 m/z was performed, followed by 0.080 s product ion scans from 230 to 1800 m/z in the 37 defined windows (3.05 sec/cycle). The collision energy for each window was calculated for 2+ charged ion at the centre of each SWATH block with a collision energy spread of 15 eV. The MS was always operated in high sensitivity mode.

### Protein identification, validation and quantification

After the LC-MS/MS, the resulting SCIEX.wiff data-files were processed by the ProteinPilot v5.0 search engine (AB SCIEX) and the Paragon algorithm (4.0.0.0, 4767) was used to search against the UniProt_mammals database with the following parameters: trypsin specificity, cys-alkylation and the search effort set to through. To avoid using the same spectral evidence for more than one protein, the identified proteins were grouped based on MS/MS spectra by the Protein-Pilot Pro Group™ algorithm, regardless of the peptide sequence assigned. The protein within each group that could explain the most spectral data with confidence was depicted as the primary protein of the group. The SCIEX.wiff data-files obtained from SWATH experiment were analysed by PeakView® (v2.1, AB SCIEX) and peaks from SWATH were extracted with a peptide confidence threshold of 95% confidence. A false discovery rate (FDR) less than 1% and 6 transitions per peptide were required to quantify one peptide. The extracted ion chromatograms were integrated and the area under the curve (AUC) was used to calculate total protein. A normalization of the calculated AUCs was done by total sum, and the sum of all areas was equalized for all the samples.

### Gene ontology and bioinformatics analysis

The bioinformatics of differentially expressed sperm proteins was manually annotated using two comprehensive bioinformatic tools, specifically UniProt KB and DAVID. The UniProt KB database (www.uniprot.org), downloaded 19/04/2018, contains 115,678,811 total entries with 47,499 entries encoded in the *Sus scrofa* taxonomy. The DAVID Database for Annotation, Visualization and Integrated Discovery (DAVID Bioinformatics Resources 6.8; https://david.ncifcrf.gov/^61,62^) integrates numerous public sources of protein annotation and, consequently, contains information from more than 1.5 million genes and more than 65,000 species.

### Statistical analysis

A mixed ANOVA test (IBM SPSS v24.0 software, IBM Spain, Madrid), using ejaculate as a random effect, was performed to evaluate the influence of the semen source (entire SRF vs EE or comparing the first 10 mL of the SRF, the remaining SRF and the post-SRF) and the boar (n = 5) on the post-thaw sperm attributes. In proteomics, the quantitative data obtained by PeakView® were analysed using MarkerView® (v1.2, AB SCIEX) and the peak areas were normalized by the sum of peak areas of all identified peptides. Principal Component Analysis (PCA) was performed to evaluate the discrimination ability of FT-sperm proteins between semen sources from Experiment 1 and 2. Then, the Multiexperiment Viewer (MeV) software (version 4.8) (http://www.tm4.org/mev.html) was used to identify changes in relative proteins abundance among FT-sperm sources. Once identified, differences in protein abundance were statistically evaluated using a Student’s t-test and ANOVA in Experiment 1 and 2, respectively. Differentially abundant proteins among FT-sperm sources were defined using an adjusted P-value < 0.01, and those with a fold change (FC) ≥ 1.50 after log2 transformation were highlighted. The explanatory ability of the resulting selected proteins in both experiments was illustrated by mean of heat maps after z-score normalization using Euclidean distances.

## Electronic supplementary material

Dataset 1

## Data Availability

All data generated in the experiments are provided in supplementary files.
